# Prostaglandin and antigestagen in pyometra bitches: vascular and stereological effect

**DOI:** 10.1530/RAF-20-0020

**Published:** 2021-04-19

**Authors:** Roberto Rodrigues da Rosa Filho, Maíra Morales Brito, Thaís Gomes Faustino, Leticia Lima de Almeida, Verônica Correia Manoel, Bruno Cogliati, Camila Infantosi Vannucchi

**Affiliations:** 1 Department of Animal Reproduction, School of Veterinary Medicine and Animal Science, University of São Paulo, São Paulo, Brazil; 2 Department of Pathology, School of Veterinary Medicine and Animal Science, University of São Paulo, São Paulo, Brazil

**Keywords:** aglepristone, angiogenesis, uterus, nitric oxide, Doppler

## Abstract

Effects of conservative treatment on uterine blood flow and morphometric findings are still unknown in bitches. Thus, this study aimed to compare uterine changes of pyometra bitches subjected to distinct modes of treatment. Pyometra bitches were assigned to: OHE (ovariohysterectomy immediately after diagnosis), Aglepristone (days 1, 2 and 8) and Associative (aglepristone treatment coupled with cloprostenol for 7 days) groups. After 9 days, bitches were ovariohysterectomized. Before surgery, uterine area was measured ultrasonographically and the uterine artery Doppler velocimetry analyzed blood flow velocity and indexes. Uterine horns were classified according to resistance index (RI) as more compromised and less compromised. Endometrial vasculature was quantitatively evaluated by color flow Doppler. Blood samples were collected to determine nitric oxide (NO) concentrations. Histological uterine structures were quantified by stereology and VEGF-A (vascular endothelial growth factor) and eNOS were (endothelial nitric oxide synthase) immunohistochemically analyzed. Aglepristone and Associative groups had lower uterine area and vascularization, and higher blood flow velocity and indexes compared to OHE group. Less compromised horn of Associative group had higher blood flow velocity compared to OHE group. Aglepristone group presented lower inflammatory infiltrate and larger uterine stroma. Associative group had lower volume density and absolute surface of endometrial cysts and lower VEGF-A expression for glandular epithelium and stromal cells. Blood NO and e-NOS immunostaining were not different among groups. In conclusion, association between aglepristone and prostaglandin is more effective in decreasing uterine vascularization and modulating uterine blood flow. Moreover, associative therapy promotes marked morphological changes.

**Lay summary:**

This research compared two medical protocols of treatment for uterine infection (pyometra) in bitches, using a hormone blocker (anti-progesterone aglepristone) solely or in association with a uterine contraction inducer (prostaglandin; associative therapy). After treatment, bitches were gonadectomized and a microscopic analysis of uterine blood vessel formation and uterine tissue elements were performed as well as uterine blood flow evaluation through Doppler ultrasonography. According to vascular resistance, uterine horns were additionally classified as more compromised and less compromised. Both treatment protocols led to reduction of uterine dimensions and vascularization, and higher blood flow compared to untreated bitches. Less compromised uterine horn of the associative treatment had higher blood flow compared to untreated bitches. The hormone blocker treatment had lower inflammatory cells and larger uterine histological structure, while associative treatment had less uterine pathological cysts and lower blood vessel formation. The associative therapy is effective in decreasing uterine vascularization and modulating uterine blood flow as well as reestablishing endometrium structure in bitches with uterine infection.

## Introduction

Cystic endometrial hyperplasia-pyometra (CEH–pyometra) syndrome is the most frequent and important endometrial disorder in unneutered bitches (Bigliardi *et al.* 2004, Smith 2006), commonly affecting females during diestrus before 10 years of age (Baithalu *et al.* 2012). CEH–pyometra is considered as an inflammatory and infectious disorder of the endometrial mucosa, leading to suppurative morphological changes, such as cystic dilation and hyperplasia of endometrial glands (Younis *et al.* 2014).

CEH–pyometra can be classified as CEH-mucometra and endometritis-pyometra, the latest characterized by a marked inflammatory reaction (De Bosschere *et al.* 2001). More recently, a high expression of inflammatory, cell proliferation and angiogenesis markers were verified in pyometra bitches, in comparison to CEH-mucometra (Veiga *et al.* 2017). Additionally, Singh *et al.* (2018) showed a higher expression of inflammatory cytokines in atrophic pyometra compared to hyperplastic pyometra. Thus, canine pyometra has its course marked by increased uterine blood flow and endometrial morphofunctional changes, along with important clinical signs that deserve special medical attention.

Ovariohysterectomy is the most recognized treatment for CEH-pyometra bitches, mainly for closed cervix pyometra or bitches with remarkable systemic changes (Veiga *et al.* 2013). However, novel therapeutic modalities, rather than surgical treatment, have been developed over the years for breeding bitches or those females for which surgery is ruled out, due to unfavorable clinical conditions or comorbidities for anesthesia (Fieni *et al.* 2014). Medical therapeutic protocols for pyometra include a natural or synthetic prostaglandin F2-α (PGF2-α) (Jena *et al.* 2013) and/or aglepristone, an antigestagen that blocks progesterone receptors (Contri *et al.* 2015), in addition to other medical combinations and supportive care.

Although conservative treatment protocols have proven efficient for pyometra bitches in a clinical point of view (Fieni 2006, Uçmak & Tek 2008, Jena *et al.* 2013, Ros *et al.* 2014, Contri *et al.* 2015, Rosa Filho *et al.* 2020), a more in-depth analysis is still lacking. Until now, no studies have attested the effectiveness of pyometra medical therapy in restoring uterine blood flow, vascular and morphological changes in bitches. Thus, we believe that medical treatments for pyometra reestablish uterine vascularization and reduce local angiogenesis, in addition to promoting structural restitution of the uterine tissue. Albeit aglepristone can neutralize the main causal hormone factor of pyometra (progesterone-induced changes of the endometrium), coupling treatment with a drug that can remove uterine content and cause luteolysis may augment the chances of therapeutic outcome in an uterine morphological prospective. Therefore, we hypothesize that the association between aglepristone and prostaglandin is a more effective conservative treatment rather than aglepristone solely.

Thus, the aim of this study was to compare the effects of two conservative protocols for pyometra therapy on uterine blood flow and angiogenesis as well as quantitative unbiased stereological uterine features of bitches.

## Materials and methods

The current study was approved by the Bioethics Committee of the School of Veterinary Medicine and Animal Science – University of São Paulo, under protocol 5997311017.

### Animals and experimental groups

For the purpose of this study, we analyzed 17 pyometra bitches, aged 4–10 years old, of different breeds and body weights (Table 1). As an inclusion criterion, all bitches presented with open-cervix pyometra, which was diagnosed by means of ultrasonographic findings (intraluminal uterine content, increased endometrial vascularization and high blood flow and low resistance of the uterine artery; Veiga *et al.* 2017). Additionally, bitches had clinical and laboratory signs, such as sanguinopurulent or purulent vaginal discharge, lethargy, inappetence, depression and dehydration; elevated white blood cell count, anemia and hyperproteinemia. Bitches with severe systemic conditions (kidney and liver dysfunction; sepsis or peritonitis) or closed cervix pyometra were considered as exclusion criteria.

**Table 1:** Descriptive information (breed, age, weight, estrous cycle phase, and parity) of bitches enrolled in the aglepristone, associative and OHE groups.

Group/breed	Age (years)	Weight (kg)	Estrous cycle	Parity
Aglepristone				
Dachshund	8	8.8	Diestrus	Nulliparous
Weimaraner	8	44	Diestrus	Nulliparous
Lhasa Apso	9	3.6	Diestrus	Nulliparous
Mixed-breed	7	11.3	Diestrus	Nulliparous
Mixed-breed	8	22.3	Diestrus	Nulliparous
Mean ± s.e.	8.0 ± 0.31^a^	18.0 ± 7.18^a^		
Associative				
Boxer	9	23	Diestrus	Nulliparous
Mixed-breed	10	16.4	Diestrus	Nulliparous
Mixed-breed	10	13.3	Diestrus	Nulliparous
Mixed-breed	4	11.4	Diestrus	Primiparous
Labrador	9	35	Diestrus	Nulliparous
Mean ± s.e.	8.4 ± 1.12^a^	19.8 ± 4.27^a^		
OHE				
Mixed-breed	8	18	Diestrus	Nulliparous
Boxer	10	24	Diestrus	Nulliparous
Mixed-breed	10	20	Diestrus	Nulliparous
Akita	15	16	Diestrus	Nulliparous
Mixed-breed	10	22	Diestrus	Nulliparous
Poodle	10	19	Diestrus	Nulliparous
Mixed-breed	15	21	Diestrus	Nulliparous
Mean ± s.e.	11.1 ± 1.12^a^	20.0 ± 1.00^a^		

^a^Indicates non-significant difference (*P >* 0.05) among groups.

The bitches were randomly assigned to three experimental groups, according to the therapeutic protocol (Table 1):

*Ovariohysterectomy (OHE) group (n = 7):* bitches immediately subjected to ovariohysterectomy after pyometra diagnosis. The OHE group served as a control group (untreated bitches), mainly for the stereological and immunohistochemical analysis.
*Aglepristone group (n = 5):* bitches subjected to s.c. injections of 10 mg/kg of aglepristone (RU46534 – Alizin® –30 mg/100 mL VIRBAC, Carros, France) once a day on days 1, 2 and 8 after pyometra diagnosis (Fieni 2006).
*Associative therapy group (n = 5):* bitches subjected to s.c. injections of 10 mg/Kg of aglepristone (Alizin® –30 mg/100 mL VIRBAC, Carros, France) once a day on days 1, 2 and 8, coupled with i.m. injections of 1 µg/kg of cloprostenol (Ovolute® – 7.5 mg/100 mL DRAG PHARMA, Santiago, Chile) once a day from days 1 to 7 after pyometra diagnosis (Fieni 2006).

aTo assure the appropriate sample size, an analysis was conducted with the SAS power and sample size 12 (SAS Institute Inc., Cary, NC, EUA). A retrospective analysis of the data indicated there was a power of 0.87, which is considered an acceptable statistical power (at least 0.8). Hence, a minimum of five dogs per group was sufficient to demonstrate significant differences in the data.

All bitches of the Aglepristone and Associative groups remained under hospitalization and intensive care from days 1 to 9. Supportive therapy was constantly provided (5-10 mL/kg/h of i.v. 0.9% saline solution, daily) as well as antibiotic therapy (10 mg/kg/day of enrofloxacin (Enromic® – 5 mg/100 mL – Microsules Laboratory– Uruguay) and 30 mg/kg/day of metronidazole (Endonidazol® – 5 mg/mL – Fresenius Kabi Brazil Ltda, Aquiraz, Ceara)). On the 9th day after pyometra diagnosis, all bitches were subjected to ovariohysterectomy.

### Analysis of serum nitric oxide (NO)

Immediately before OHE, blood samples were collected from the bitches by puncturing the right or left jugular vein and transferred to evacuated tubes without anticoagulant. Samples were centrifuged for 10 min at 1500***g***. Serum was drawn off and stored at −20°C until it was analyzed.

Serum nitric oxide concentrations were indirectly determined according to the protocol described by Maurer *et al.* (2015). Deproteinization process was performed by adding 20 μL of 30% zinc sulfate (diluted in 1N hydrochloric acid) to 400 μL of serum. Mixture was then centrifuged at 500 ***g*** for 15 min and the supernatant was separated. Subsequently, 50 μL of the deproteinized sample and 50 μL of vanadium chloride (8 mg/mL, diluted in 1N hydrochloric acid) were added to a microplate, which was kept in the absence of light until quantification. For the Griess reaction, 25 μL of 2% sulfanilamide was added, diluted in 5% phosphoric acid and incubated for 10 min. Then, 25 μL of 0.2% N-(1-naphthyl) ethylene diamine (diluted in deionized water) was added and the sample was incubated at 37°C for 20 min. Nitric oxide was quantified spectrophotometrically at a wavelength of 540 nm (Biotek EL808®) and results were described as micromoles per liter.

For each microtiter plate, a standard curve of nitrite was also performed in duplicate for reference purpose. Different known concentrations of a sodium nitrate solution diluted in deionized water (from 100 to 0 mM, reducing half the concentration every other well) were used to build a linear equation, by which the concentration of nitrite in each serum sample was calculated.

### Uterine ultrasonographic evaluation

Ultrasonographic examination was carried out immediately before OHE in all experimental groups,that is, on the day of pyometra diagnosis in the OHE group and on day 9 after treatment onset in the Aglepristone and Associative groups. Transabdominal ultrasound evaluation was performed always by the same operator with the use of a 5 MHz micro convex transducer (Mindray® ultrasound M5Vet) in a right and left lateral recumbence. Cross-sections of the uterine horns were made in B-mode as an ellipse, due to the difference between horizontal and vertical axis of the uterine horn having intraluminal content. In order to exclude the subjectivity of such examination, we used only two axes and the area was calculated by the following formula: *R*1 × *R*2 × π, considering *R*1 = uterine width/2 and *R*2 = uterine height/2 (Silva 2014).

For the qualitative evaluation of the endometrial vasculature, scanning of uterine horns was preferably carried out in cross-section in B-mode, followed by color flow Doppler. A score from 1 to 3 was adopted, considering score 1 as the minimum degree of endometrial vasculature and score 3 the maximum degree, according to classification proposed by Veiga *et al.* (2017).

Doppler velocimetry of the right and left uterine arteries was performed in longitudinal section at the lateral region of the uterine body, using the bladder as an acoustic window. Pulsed-wave Doppler was used to characterize the waveform and to measure the overall blood flow of the uterine artery, by analyzing the following vascular parameters: blood flow velocity (peak systolic velocity (PS), end diastolic velocity (ED) and time average maximum velocity (TAMAX)) and indexes (resistance index (RI), pulsatility index (PI) and peak systolic : diastolic velocity (S/D)). Resistance index of the uterine artery was also used to classify the uterine horns as the more compromised and less compromised,that is,the uterine horn containing the artery with the lowest RI value (Veiga *et al.* 2017) was considered the more compromised and, conversely, the contralateral uterine horn was classified as the less compromised.

### Uterine stereological analysis

After hysterectomy, the uterine horns were separated from the uterine corpus and each uterine horn volume was measured by the immersion method (Scherle 1970). In addition, the weight of each uterine horn was measured by digital scale (wet weight, g) (SF-400®) in order to determine the uterine horn density (g/cm^3^). Sequentially, uterine horns were sectioned into 2 cm fragments and selected by applying a multistage systematic uniform random (SUR) design, that is, one of the every other two fragments was selected and collected. The fragments were then longitudinally and transversely sectioned alternately, resulting in four sub-fragments, sampled again by SUR. Thus, the sampling procedure resulted in one to four sets of SUR fragments per uterine horn, and three to five fragments were sampled in each set.

The tissue samples were fixed in 10% buffered formaldehyde solution for at least 48 h and subsequently stored in 70% alcohol solution for later inclusion in paraffin, dehydration and diaphanization. Sections (5-μm) were randomly selected and submitted to deparaffinization and stained with hematoxylin-eosin (Veiga *et al.* 2017).

At least ten random fields of view from each uterine section were photomicrographed using OLYMPUS BX60® microscope with a Zeiss AxioCam HRc® digital camera, equipped with a 4× objective lens in order to view each section entirely using Zen Blue 2.6® software (Carl Zeiss). Volume fraction of each uterine region (myometrium (M), endometrium (End) and cyst (C)) was estimated by the point-counting method (Howard & Reed 2005, Salinas *et al.* 2017) provided by ImageJ® software. On specimen scale, the test grid had a distance in pixels of 0.3075 pixels/μm. Test grid points falling into each region (P^region^) and into the entire uterine horn (P^horn^) were counted, and volume fractions (Vv, %) within uterine horn were estimated using the following equation: Est Vv = ∑P^region^/∑P^horn^. The total volume of each region (V^region^, cm^3^) was estimated by multiplying the volume fraction (Vv) by the corresponding uterine volume (V^horn^).

The volume fraction of each endometrial structure (cysts (Cy), stroma (S), glands (G), inflammatory infiltrate (I), luminal epithelium (L), vessels (V) and edema (E)) was estimated as previously described. However, a 20× objective lens was used to obtain four to ten fields of the endometrium. On specimen scale, the test grid had a distance of 1.5383 pixels/inch. Test grid points falling into each structure (P^structure^) and into the entire endometrium (P^End^) were counted, and volume fractions (Vv, %) of each structure within the endometrium were estimated using the following equation: Est Vv = ∑P^structure^/∑P^End^. At least 200 to 300 points were counted. The total volume of each endometrial structure (V^structure^) was estimated by multiplying the volume fraction (Vv) by the corresponding endometrial volume (V^End^).

Surface density of glands (G), luminal epithelium (L), vessels (V) and cysts (Cy) was obtained using a grid of cycloid arcs superimposed on ten random fields photographed using a 20× objective lens (Baddeley *et al.* 1986). Intersections between test arcs and the boundary traces of the endometrial structures were counted and summed over all fields for each endometrium. The following equation was used to estimate the density of each surface (Sv, cm^−1^) from endometrial structures: Est Sv = 2 × ∑I^surf^/(lp × ∑P^End^); ∑I^surf^ is the number of interceptions between cycloid arcs and the compartment surface, lp is the length of the test line (81.64 µm on the specimen scale) associated with each point, and ∑P^End^ is the number of points hitting the reference space (endometrium).

The absolute surface area (S^surf^, cm^4^) was obtained by multiplying the surface density by endometrium volume.

### Immunohistochemical reactions for VEGF-A (vascular endothelial cell growth factor) and eNOS (endothelial nitric oxide synthase)

For immunohistochemical purpose, two random samples were analyzed per uterine horn (right and left) from each bitch. All immunohistochemical reactions were performed in duplicate.

Immunohistochemical reaction was performed with VEGF-A primary antibody (ab1316 Abcam®, canine cross reactivity according to Supplementary Table 1, see section on supplementary materials given at the end of this article) in 1:300 dilution with antigen retrieval performed with citrate buffer (pH 6.0) and subsequently in secondary antibody (Novolink^TM^ Max Polymer Detection System, Rabbit, Novocastra, Leica Biosystems®). For the eNOS primary antibody (ab5589 Abcam®, canine cross reactivity according to Supplementary Table 1), the reaction was performed with citrate buffer (pH 6.0) in 1:50 dilution and posteriorly in secondary antibody (Universal kit LSABTM + kit/HRP, Rb/Mo/Goat, Dako®). All immunohistochemical reactions were accompanied by a positive control (cerebellum for VEGF-A antibody; kidney for e-NOS antibody) and negative control (tissue belonging to the OHE group in which the primary antibody was not applied) (Supplementary Figs 1 and 2).

**Figure 1:**
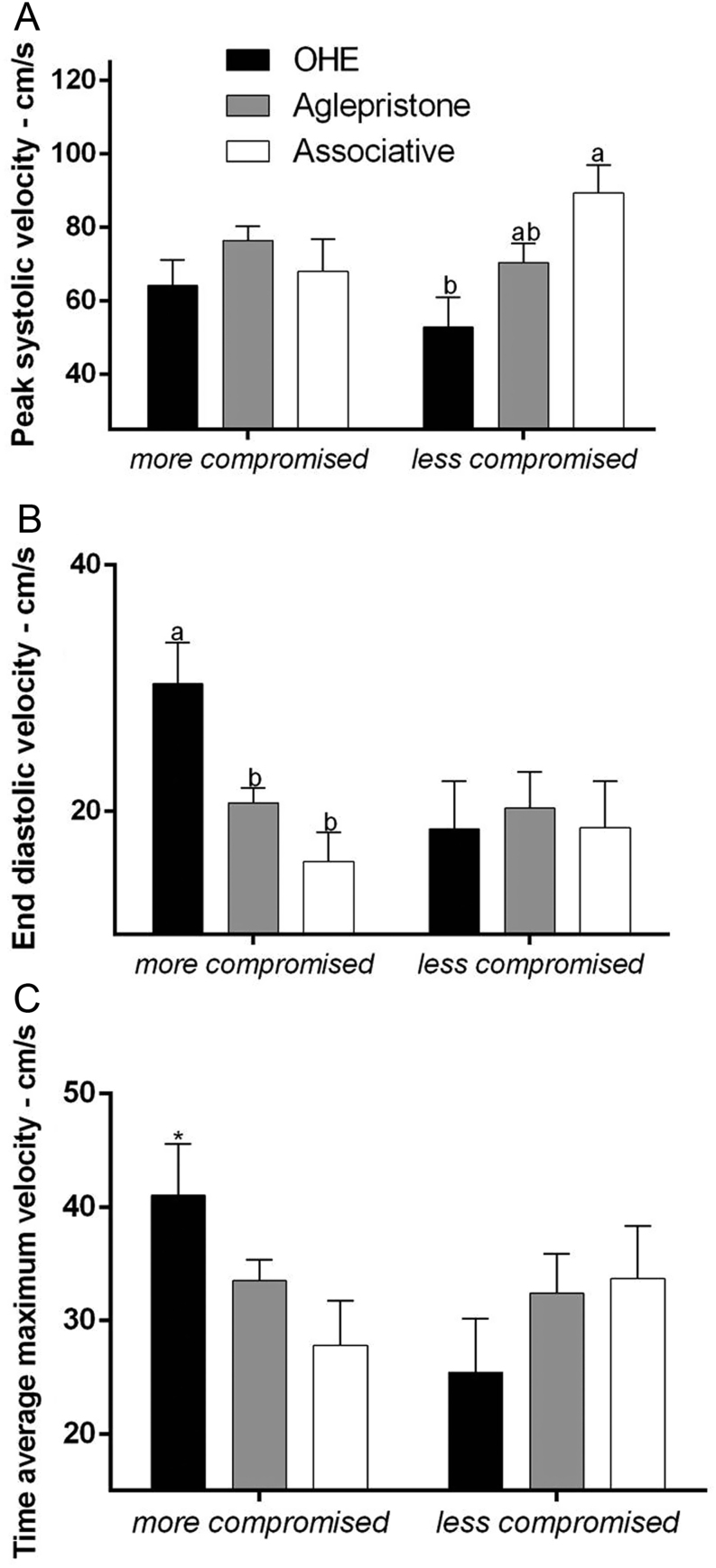
Doppervelocimetry of the more or lesscompromised uterine artery. (A) Peak systolic blood velocity (cm/s), (B) end diastolic blood velocity (cm/s) and (C) time average maximum blood velocity (cm/s) in the OHE, Aglepristone and Associative groups. *Indicate significant differences between moreand less compromised uterine horn in the same group. ^a–b^Values with different letters differ significantly between groups (*P* < 0.05).

Analysis was performed by two operators in an OLYMPUS BX60® microscope with Zeiss AxioCam HRc® digital camera and evaluated with a 20× objective lens in the Zen Blue 2.6® software(Carl Zeiss). For the analysis of VEGF-A, the endometrial tissue was divided into three cell clusters analyzed separately: luminal epithelium, glandular epithelium and cells present in endometrial stroma (Veiga *et al.* 2017). For e-NOS, the uterine tissue was divided in five cell clusters: glandular epithelium, luminal epithelium, cells present in endometrial stroma, endometrial vascular endothelium and myometrial vascular endothelium. Analysis was made using the relationship between two variables: percentage of stained area (0: negative to 5%, 1: 5–33%, 2: 33–66%, 3: above 66%) and staining intensity (0:negative, 1:weak, 2:moderate, 3:intense). Based on these variables, a score was defined by the following calculation: immunohistochemical score = percentage of stained area × staining intensity (Veiga *et al.* 2017).

### Statistical analysis

All data were evaluated using the SAS System for Windows version 9.3 (SAS Institute Inc., Cary, NC, USA). The data were analyzed using one-way ANOVA. Differences between groups were analyzed using parametric tests, according to the residual normality (Gaussian distribution) and variance homogeneity. Whenever one of these assumptions was not valid, data were transformed, according to the Guided Data Analysis Procedure of the SAS System for Windows. When normality was not obtained, the NPAR1WAY procedure of nonparametric ANOVA was used.

For the following variables: uterine area, qualitative evaluation of the endometrial vasculature and blood NO, comparisons among groups were evaluated by ANOVA test. For PW Doppler ultrasonography, immunohistochemistry and stereology, data were evaluated according to a double interaction: groups (OHE vs Aglepristone vs Associative) and degree (more compromised vs less compromised) considering significant interactions as *P* < 0.1, which is considered an adequate interaction *P*-value for factorial experiments (Cavalcanti *et al.* 2010). B-mode ultrasound variables were not subjected to an interaction analysis of groups and degrees due to a technical difficulty in distinguishing exactly the right and left fulfilled uterine horn in an abdominal assessment. If no significant interaction occurred (*P* > 0.1), ANOVA/LSD tests were used to compare between groups (combining all degrees) and Student's *t-*/Wilcoxon-test between degrees (combining all groups). Bitches’ age and weight were also compared among groups by ANOVA/Tukey Test.

Variables were also submitted to Spearman correlation analysis, considering all animals, regardless the experimental group.

Results are expressed as mean ±s.e. and statistical differences were considered significant if *P* < 0.05.

## Results

In total, the 17 pyometra bitches were aged from 4 to 15 years. The OHE group had a mean age of 11.1 ± 1.12 years, while the Aglepristone and Associative groups aged, respectively, 8.0 ± 0.31 and 8.4 ± 1.12 years, without significant difference (*P* > 0.05) among groups (Table 1). According to body weight, the recruited bitches weighted from 3.6 to 44 kg, that is, small breed bitches (<10 kg; 2/17), medium breed bitches (between 10 and 25 kg; 13/17) and large breed bitches (> 25 kg; 2/17). Bitches of the OHE group had a mean weight of 20.0 ± 1.00 kg, whereas the Aglepristone and Associative groups weighted, respectively, 18.0 ± 7.18 and 19.8 ± 4.27 kg without significant difference (*P* > 0.05) among groups (Table 1).

### Uterine ultrassonographic examinations

Before the onset of treatment, all bitches had similar Doppler ultrasonographic parameters. No differences (*P* > 0.05) among groups occurred regarding uterine artery hemodynamic variables at the beginning of the experiment (Supplementary Table 2).

**Table 2:** Mean s.e. (± s.e.) of immunostaining score of uterine endothelial nitric oxide synthase (e-NOS) and blood concentration of nitric oxide in the OHE, Aglepristone, and Associative groups.

	OHE group	Aglepristone group	Associative group
e-NOS glandular epithelium	3.17 ± 0.50	2.6 ± 0.69	2.4 ± 0.43
e-NOS luminal epithelium	1.79 ± 0.41	1.33 ± 0.44	1.3 ± 0.42
Stromal cells	5.29 ± 0.7	7 ± 0.86	5.6 ± 0.76
e-NOS endometrium vessels	4.31 ± 0.66	3.78 ± 0.52	3.9 ± 0.78
e-NOS myometrium vessels	3 ± 0.61	3.62 ± 1.22	3.1 ± 0.84
Nitric oxide (μmol/L)	163.75 ± 40.14	191.25 ± 98.61	194.62 ± 52.80

According to our previous results, all bitches had histological cystic endometrial hyperplasia (CEH) and compatible uterine blood flow changes (Rosa Filho *et al.* 2020). Uterine area was higher in the OHE group on day 0 (7.03 ± 1.75 cm^2^) compared to the Aglepristone group (2.38 ± 0.80 cm^2^) and Associative group (2.77 ± 0.45 cm^2^) on day 9 after onset of treatment. Endometrial vascularization score was lower in the Aglepristone group (1.6 ± 0.2) and the Associative group (1.4 ± 0.2), compared to the OHE goup (3 ± 0).

There was a significant interaction between groups and degrees for the uterine artery peak systolic velocity (*P* = 0.07), end diastolic velocity (*P* = 0.07) and TAMAX (*P* = 0.07). Data on the ultrasound examination of pyometra bitches on day 1 (before treatment in the aglepristone and associative groups) are shown by Rosa Filho *et al.* (2020). In the evaluation of blood flow of the less compromised uterine horn, peak systolic velocity (PS) was higher in the Associative group (89.49 ± 7.57 cm/s), compared to the OHE group (52.84 ± 8.11 cm/s) (Fig. 1A). Conversely, for the more compromised uterine horn, end diastolic velocity (ED) was lower in both the Aglepristone group (20.69 ± 1.20 cm/s) and the Associative group (15.93 ± 2.39 cm/s), in comparison to the OHE group (30.35 ± 3.36 cm/s) (Fig. 1B). For the OHE group, the more compromised uterine horn had a higher time average maximum velocity (TAMAX) compared to the less compromised uterine horn (Fig. 1C). No statistical difference between horns was verified for PS and ED variables. Moreover, for the more compromised uterine horn, PS and TAMAX were not different between groups. For the less compromised uterine horn, ED was not different between groups.

**Figure 2:**
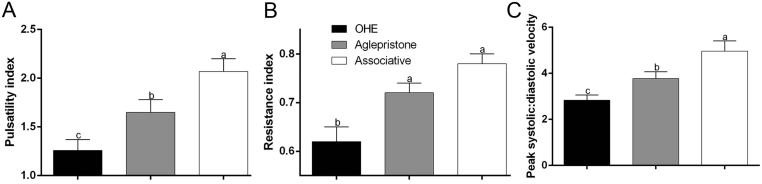
Blood flow indexes of the uterine artery. (A) Pulsatility index, (B) resistance index and (C) peak systolic: diastolic velocity in the OHE, Aglepristone and Associative groups. ^a^ ^–c^Values with different letters differ significantly between groups (*P* < 0.05).

The Associative group had higher pulsatility index and peak systolic: diastolic (S/D) compared to the Aglepristone group, which was higher than the OHE group (Fig. 2A and C). On the other hand, resistance index (RI) was lower in the OHE group compared to both the Aglepristone group and Associative group (Fig. 2B). No differences between degrees of uterine artery were verified for the blood flow indexes.

### Uterine stereological examination

There was a significant interaction between groups and degrees for the uterine horn density (*P* = 0.07). For the more compromised uterine horn of the Aglepristone goup, lower (*P* = 0.004) uterine horn density (0.98 ± 0.02 g/cm^3^) was verified in comparison to both the Associative group (1.05 ± 0.01 g/cm^3^) and the OHE group (1.05 ± 0.02 g/cm^3^). Moreover, in the Aglepristone group, the more compromised uterine horn had lower (*P* = 0.03) specific density (0.98 ± 0.02 g/cm^3^) in comparison to the less compromised uterine horn (1.03 ± 0.01 g/cm^3^).

There was no statistical difference among groups for the volume fraction (Vv) and volume (V) of uterine regions (myometrium, endometrium, cysts) as well as between the less andmore compromised uterine horn.

**Figure 3:**
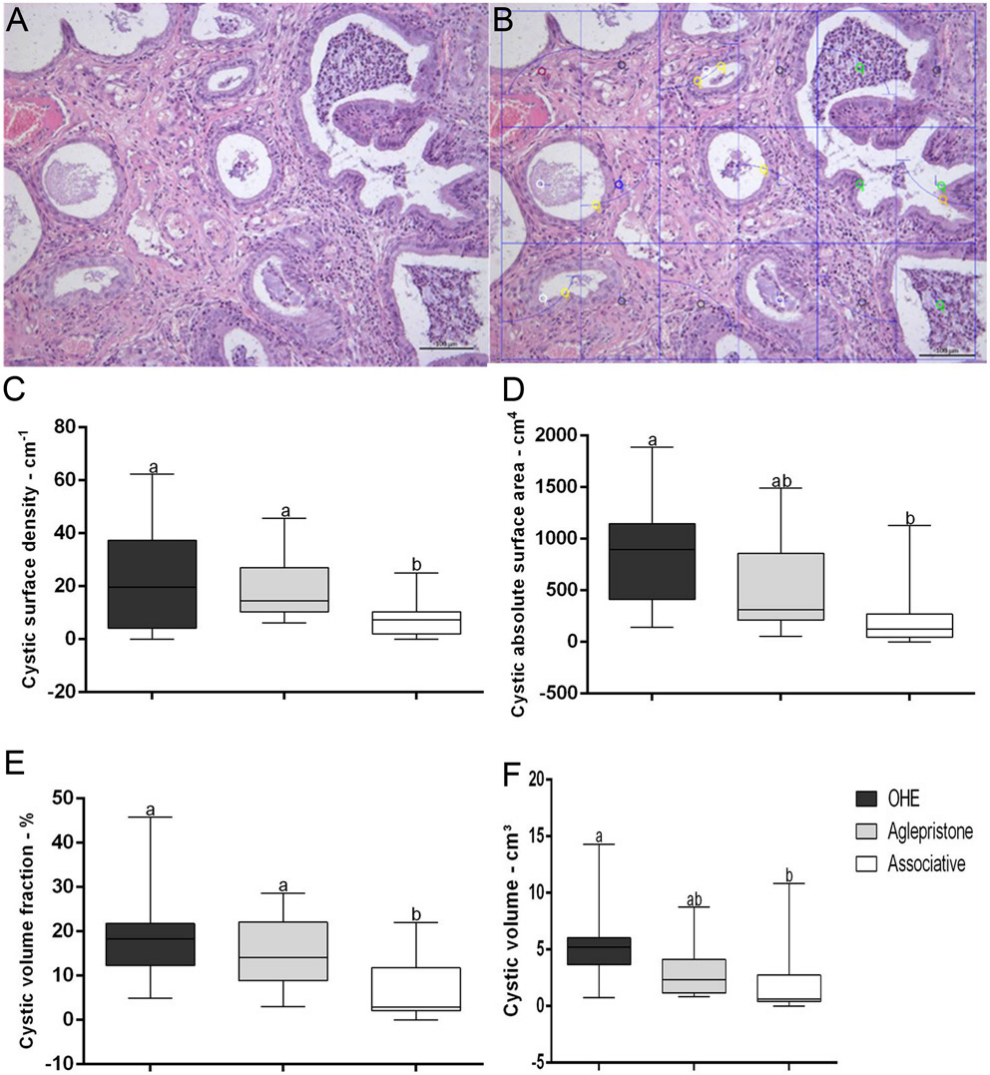
(A) Histological image of contact surface of endometrial structures.Hematoxylin and eosine stain 20×. (B) Point-counting method with test grid and grid of cycloid arcs for the quantitative unbiased stereology of endometrial structures. Hematoxylin and eosine stain 20×. (C) Cystic surface area (Sv^Cy^). (D) Cystic absolute surface area (S^Cy^). (E) Cystic volume fraction (Vv^Cy^) and (F) cystic volume (V^Cy^) in the OHE, Aglepristone and Associative groups. ^a–b^Values with different letters differ significantly between groups (*P* < 0.05).

The Aglepristone group had lower (*P* = 0.03) endometrial inflammatory infiltrate volume fraction (Vv^I^; 35.33 ± 4.74%) in comparison to both the OHE group (48.35 ± 4.08%) and the Associative group (52.28 ± 3.59%). In addition, the Aglepristone group had a higher (*P* = 0.006) endometrial stroma volume fraction (Vv^S^; 17.39% (10.38;20.95)) compared to the OHE group (4.32% (2.78;11.11)). Cystic surface density (Sv^Cy^) and volume fraction (Vv^Cy^) were lower (*P* = 0.002 and *P* = 0.01, respectively) in the Associative group in comparison to both the Aglepristone group and the OHE group (Fig. 3C and E). The Associative group had lower cystic absolute surface area (S^Cy^) and volume (V^Cy^) compared to the OHE group (*P* = 0.03 and *P* = 0.05, respectively) (Fig. 3D and F). The less compromised uterine horn had higher edema volume fraction (Vv^E^; 4.85% (1.5;10.60)) and volume (V^E^; 0.85 cm^3^ (0.42;1.65)), in comparison to the more compromised uterine horn (1.44% (0;2.94) and 0.46 cm^3^ (0;0.69); *P* = 0.02 and *P* = 0.04, respectively). No statistical difference was observed for the remaining stereological variables.

### IHC reactions for VEGF-A and e-NOS – serum NO analysis

**Figure 4:**
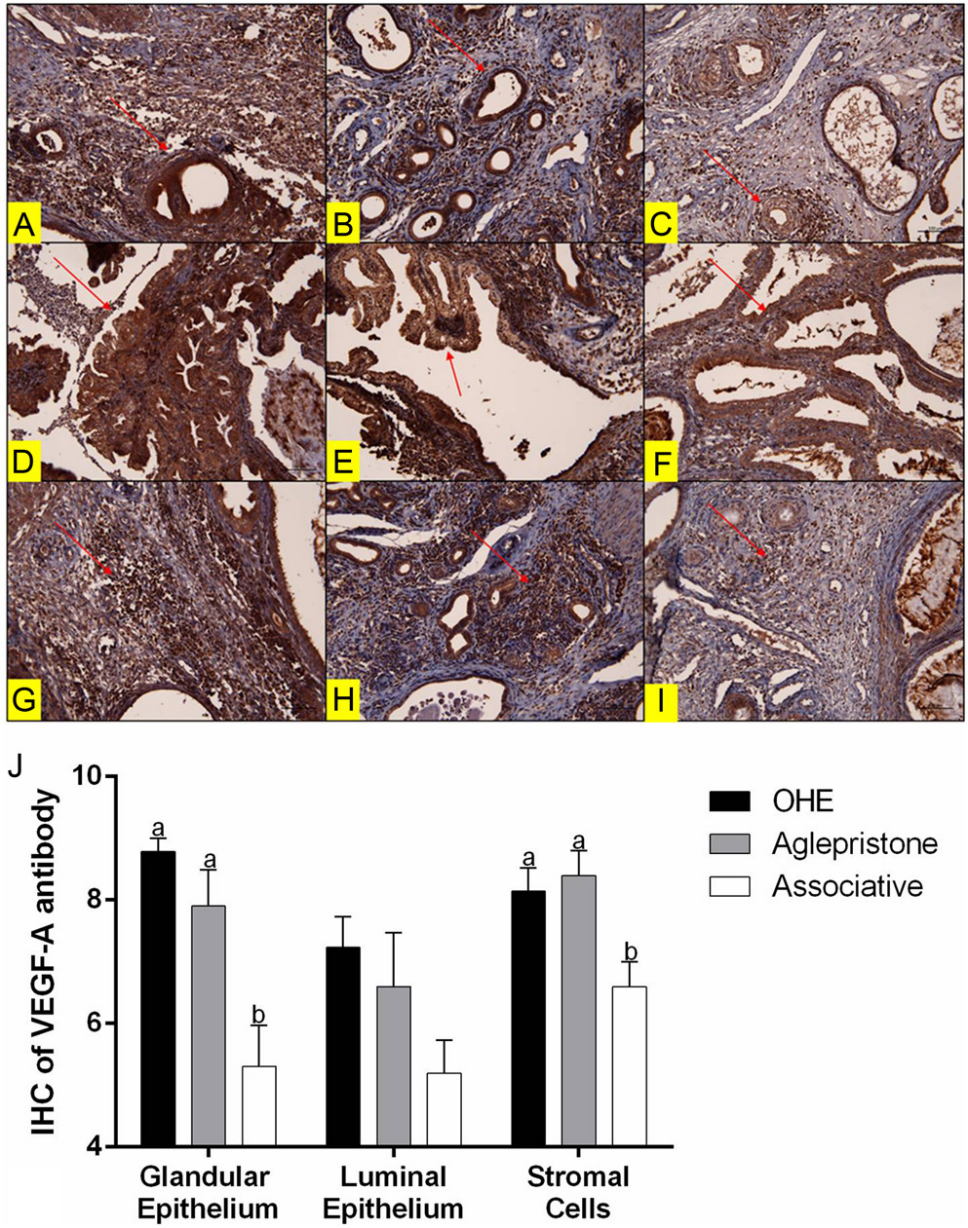
Cytoplasmatic immunostaining of VEGF-A in the glandular epithelium (arrows) in the (A) OHE group, (B) Aglepristone group and (C) Associative group. VEGF-A immunostaining in the luminal epithelium (arrows) in the (D) OHE group, (E) Aglepristone group and (F) Associative group. VEGF-A immunostaining in stromal cells (arrows) in the (G) OHE group, (H) Aglepristone group and (I) Associative group 200×. (J) VEGF-A immunostaining score according to the uterine cell cluster in the OHE, Aglepristone and Associative groups. ^a–b^Values with different letters differ significantly between groups (*P* < 0.05).

The staining intensity of VEGF-A in the glandular epithelium and cells of the endometrial stroma was lower in the Associative group compared to the others groups (Fig. 4). No difference between the less and more compromised uterine horns was observed for the immunohistochemical analysis of VEGF-A.

We were not able to demonstrate any differences among groups or between degrees (less and more compromised uterine horns) for the immunohistochemical analysis of e-NOS or blood NO (Table 2). Data on blood NO of pyometra bitches on day 1 (before treatment in the aglepristone and associative groups) are shown by Rosa Filho *et al.* (2020).

### Correlation analysis

Regardless of the experimental group, the end diastolic velocity of the uterine artery had a positive correlation (r = 0.35; *P* = 0.04) with cystic surface density (Sv^Cy^). Negative correlation between uterine artery PI and absolute cystic surface area (S^Cy^; r = −0.45; *P* = 0.006), cystic volume (V^Cy^; r = −0.41; *P* = 0.01) and cystic surface density (Sv^Cy^; r = −0.53; *P* = 0.001). Uterine artery RI had a negative correlation with absolute cystic surface area (S^Cy^; r = −0.51; *P* = 0.001), cystic volume (V^Cy^; r = −0.45; *P* = 0.007), cystic surface density (Sv^Cy^; r = −0.59; *P* = 0.0002) and VEGF-A immunostaining in endometrium glandular epithelium (r = −0.35; *P* = 0.04). Peak systolic : diastolic velocity of the uterine artery had a negative correlation with absolute cystic surface area (S^Cy^; r = −0.51; *P* = 0.002), cystic volume (V^Cy^; r = −0.46; *P* = 0.006), cystic surface density (Sv^Cy^; r = −0.6; *P* = 0.002) and VEGF-A immunostaining in endometrium glandular epithelium (r = −0.36; *P* = 0.04).

Endometrial inflammatory infiltrate volume (V^I^) positively correlated with absolute cystic surface area (S^Cy^; r = 0.64; *P* < 0.0001) and cystic volume (V^Cy^; r = 0.46; *P* = 0.006). Additionally, positive correlation occurred between VEGF-A immunostaining in the glandular epithelium with cystic volume fraction (Vv^Cy^; r = 0.43; *P* = 0.01), cystic volume (V^Cy^; r = 0.38; *P* = 0.02) and cystic surface density (Sv^Cy^; r = 0.34; *P* = 0.04). VEGF-A immunostaining in stromal cells negatively correlated with endometrial stroma volume (V^S^; r = −0.34; *P* = 0.05) and endometrial stroma volume fraction (Vv^S^; r = −0.37; *P* = 0.03).

## Discussion

The present study aimed to evaluate possible changes in uterine vascularization, blood flow and angiogenesis as well as histeromorphology of medically treated bitches for pyometra. Unfortunately, for ethical reasons, we could not have left pyometra bitches untreated until 9 days after the diagnosis, in order to perform the OHE at the same time-point as treated bitches. Therefore, the different day the OHE (control) group was assessed compared to the treatment groups is an inevitable limitation of this study.

According to De Bosschere *et al.* (2001), an histological classification of pyometra includes a marked uterine stromal invasion of polymorphonuclear cells, associated or not with endometrial cystic formation. In our study, bitches were clinically diagnosed with pyometra but the uterine stereology demonstrated that the endometrium fraction volume of the OHE group (39.06 ± 1.85%) resembled the reported results of healthy bitches (35%; (Salinas *et al.* 2017)), indicating that pyometra does not change the volume of uterus layers. On the other hand, our histological findings also revealed endometrial cysts (CEH), which are important structures of the CEH-pyometra pathogenesis (Dow 1959). In addition, there was a positive correlation between stereological percentage of cysts and end diastolic velocity (ED) and infiltrate inflammatory volume as well as negative correlation with uterine artery blood flow indexes (PI, RI and S/D). Moreover, the more compromised uterine horn of untreated bitches had higher time average maximum velocity of the uterine artery, suggesting an additional vascular modulation at the focal uterine area in which a noteworthy inflammation occurred. Taking these data together, we suggest that the occurrence of endometrial cysts is capable of indirectly modulating uterine blood flow, as the amount of endometrial cysts was related to infectious/inflammatory development. Thus, the intensity of cystic formation acts imperatively on uterine blood flow. These findings should be taken into account while performing an uterine Doppler ultrasound of pyometra bitches in association with B-mode ultrasound.

Expectedly, we observed lower uterine dimensions (area) in the conservative therapy groups compared to the OHE group, showing that the medical treatment, coupled with adequate supportive care, can effectively reduce uterine area. This result can also be corroborated by the lower endometrial vasculature score in medically treated bitches, in comparison to the OHE group. However, it is important to point out that irrespective of the noticeable clinical improvement of treated bitches, endometrial vascularization was similar to CEH-mucometra, previously described by Veiga *et al.* (2017), and differed from uterine vascularization of diestrous bitches (Veiga *et al.* 2017). Thus, our medical treatment protocols for pyometra have contributed to the reestablishment of endometrial vascularization but still not achieving a through physiological condition. Therefore, based on such vascular parameter, further studies should be undertaken performing a long-term investigation of uterine changes until anestrous, in order to certify that uterine vascular regeneration of pyometra treated bitches occurs in an extended manner rather than our therapy time-period.

Comparing medical treatments in the present experiment, lower blood flow of the uterine artery (higher PS, PI, RI and S/D) was observed in the Associative group, suggesting greater influence of PGF2-α in association with aglepristone on uterine blood flow features. In fact, prostaglandin has a vasoconstriction action (Nakano & Cole 1969), which in association with local inflammatory reduction, led to significant decrease in uterine blood flow after pyometra treatment. Moreover, our dopplervelocimetric results (ED, PI, RI and S/D) were similar to those reported previously for CEH-mucometra bitches (Batista *et al.* 2015, Veiga *et al.* 2017). Thus, we may infer that the additional use of PGF2-α for pyometra treatment contributed significantly to the reduction of uterine inflammation, although not completely diminishing endometrial hyperplasia in our therapy timespan.

In relation to endometrial integrity, the Aglepristone group had higher percentage of uterine stromal area (approximately 18%), whereas the OHE group presented only 4% of uterine stroma. Additionally, only aglepristone treated bitches had significant decrease in inflammatory cell infiltrate within the uterine stroma as well as lower specific density in the more compromised uterine horn (compared to the OHE Group). The negative correlation between the volume fraction of endometrial stroma and VEGF-A immunostaining in stromal cells helps the understanding of a significant endometrial morphologic change. Thus, aglepristone treatment favored a remarkable uterine restructuring, despite an ongoing morphological imbalance of the endometrium in comparison to healthy bitches. We may imply that the use of PGF2-α in the associative protocol further stimulated an acute inflammatory cascade and leukocyte migration (Ricciotti & Fitzgerald 2011), thus not contributing to thoroughly reduce the percentage of inflammatory infiltration within endometrial stroma, in comparison to the solely use of aglepristone.

In the present study, a decrease in uterine cystic volume and surface occurred only after the associative therapy, which can be an important response to the uterine contraction stimuli of PGF2-α. Additionally, we observed a positive correlation between the intensity of cystic formation and glandular epithelium expression of VEGF-A and only the associative therapy had decreased immunostaining for VEGF-A in comparison to the OHE Group. These findings not only corroborate the coupled influence of endometrial cysts and inflammatory process on uterine angiogenesis but also attest the effectiveness of the associative treatment with PGF2-α in restoring uterine blood flow. In fact, Takasaki *et al.* (2010) showed that increased uterine vascular resistance could prevent the glandular tissue from developing and decrease VEGF-A expression. In our study, VEGF-A expression had a negative correlation with uterine artery blood flow indexes, reinforcing that the angiogenesis decrease after associative therapy contributed to diminish uterine blood flow in pyometra bitches.

Despite the observed effectiveness of pyometra conservative therapy in modulating uterine blood flow, we failed to demonstrate differences on serum NO concentration and e-NOS uterine expression among experimental groups. It is, thus, possible to infer that nitric oxide is not the main vasodilator involved in the uterine blood flow changes of pyometra. Hence, other vasodilator agents can be related to the pathogenesis and infectious process of pyometra in bitches. In fact, according to Barbagallo *et al.* (2001), progesterone acts directly as a vasodilator hormone. In addition, the increased uterine expression of Toll-like 2 and 4 receptors during pyometra course (Silva *et al.* 2010) leads to subsequent secretion of local inflammatory cytokines, such as cycloxygenase-2 and prostaglandin E_2_ (Silva *et al.* 2012), the latest considered an important uterine vasodilator in women (Sastry *et al.* 1997). Therefore, both progesterone and PGE_2_ should be considered as vasodilator agents in pyometra bitches and should be further investigated more accurately in future experiments.

In conclusion, both conservative protocols were effective in decreasing endometrial vascularization, uterine blood flow, uterine unbiased stereology (endometrial cysts and inflammatory infiltration) and increase uterine stromal integrity in pyometra bitches. However, therapy with aglepristone in association with PGF2α reduced pyometra angiogenesis more effectively, demonstrating its effectiveness as pyometra conservative therapy. Until now, no studies have been performed to in-depth compare distinct treatment protocols for pyometra in bitches, considering uterine blood flow and Doppler velocimetry, morphological features and angiogenesis. Therefore, innovative results presented here can help in underlining a successful approach for the treatment of pyometra in dogs.

## Supplementary Material

Supplementary Figure 1. Immunostaining for VEGF-A (A) Negative control in the canine cerebellum, (B) Positive control in the canine cerebellum, (C) Negative control in the canine uterus, (D) Positive control in the canine uterus. 200x.

Supplementary Figure 2. Immunostaining for eNOS (A) Negative control in the canine kidney, (B) Positive control in the canine kidney, (C) Negative control in the canine uterus, (D) Positive control in the canine uterus. 200x.

Supplementary Table 1. VEGF-A and eNOS antibody characteristics and cross reactivity.

Supplementary Table 2. Mean and standard error (X±SE) of the uterine artery

## Declaration of interest

The authors declare that there is no conflict of interest that could be perceived as prejudicing the impartiality of the research reported.

## Author contribution statement

R R R F, M M B, T G F and L L A carried out the study. V C M and B G analyzed data. R R R F wrote the manuscript with support from C I V. Moreover, C I V conceived the original idea and supervised the experiments.
