# Supplementary figures and images for: Prostaglandin and antigestagen in pyometra bitches: vascular and stereological effect
Source: Reprod Fertil. 2021 Apr 19;2(2):95–105. doi: 10.1530/RAF-20-0020 (PMC8812451; doi:10.1530/RAF-20-0020)

**A**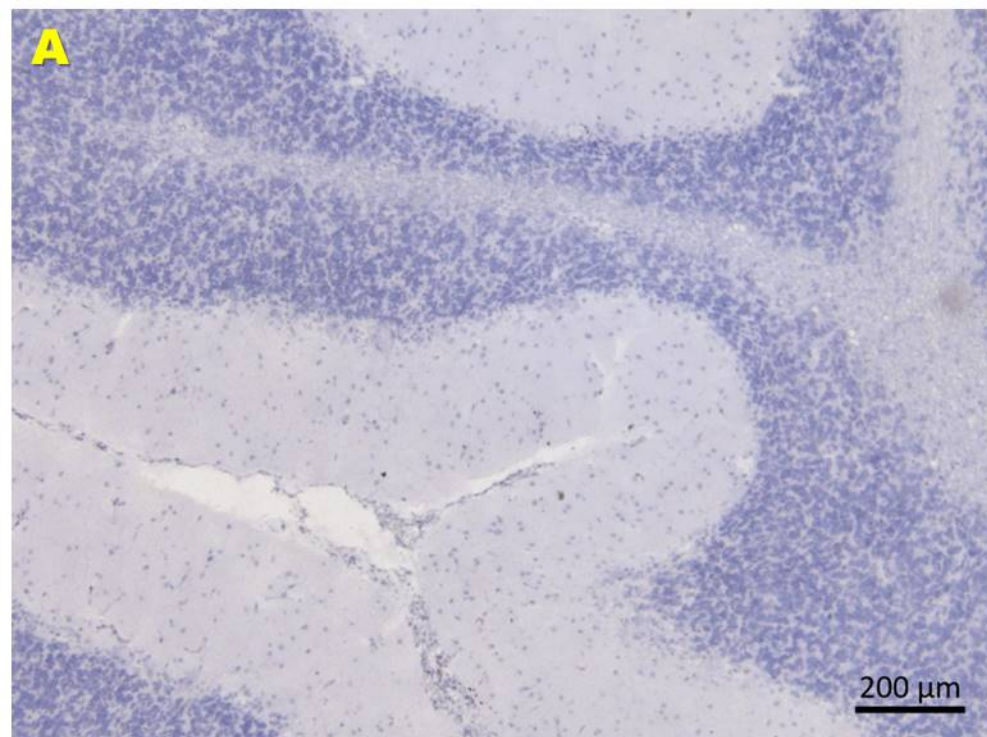**B**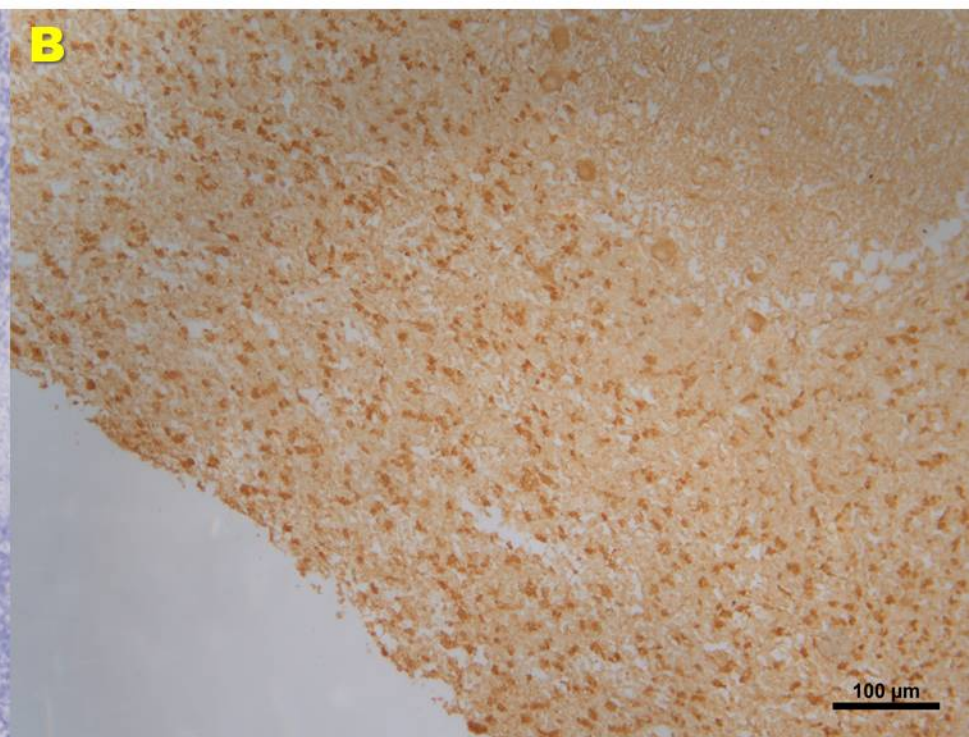**C**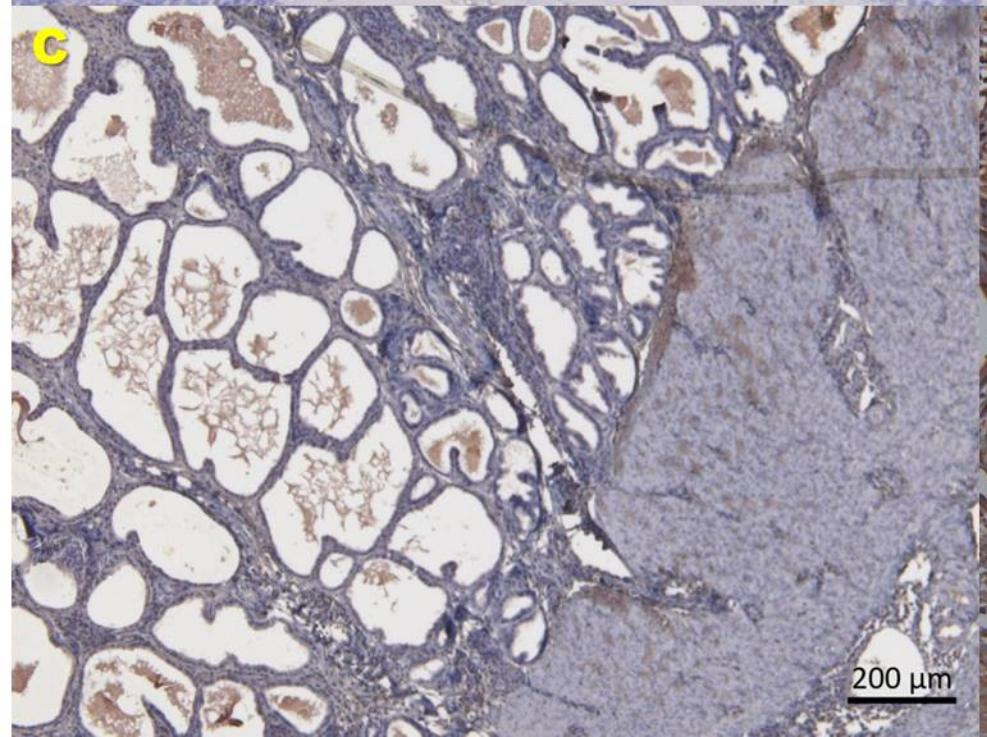**D**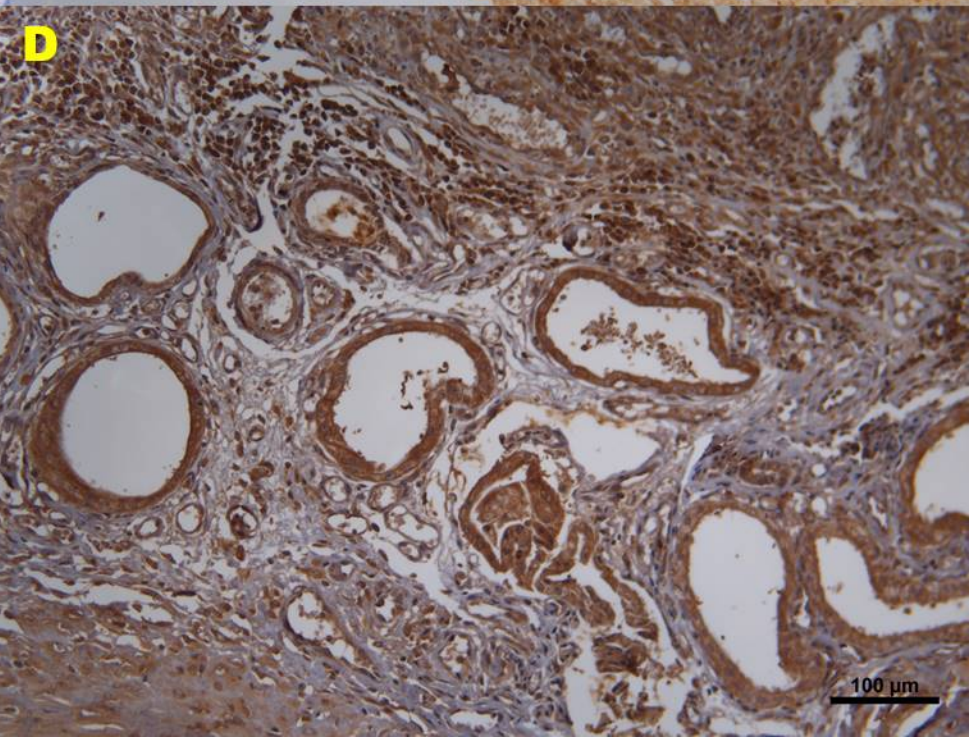

Supplement: Supplementary Figure 1. Immunostaining for VEGF-A (A) Negative control in the canine cerebellum, (B) Positive control in the canine cerebellum, (C) Negative control in the canine uterus, (D) Positive control in the canine uterus. 200x. [file supplementary_figure_1.pdf]

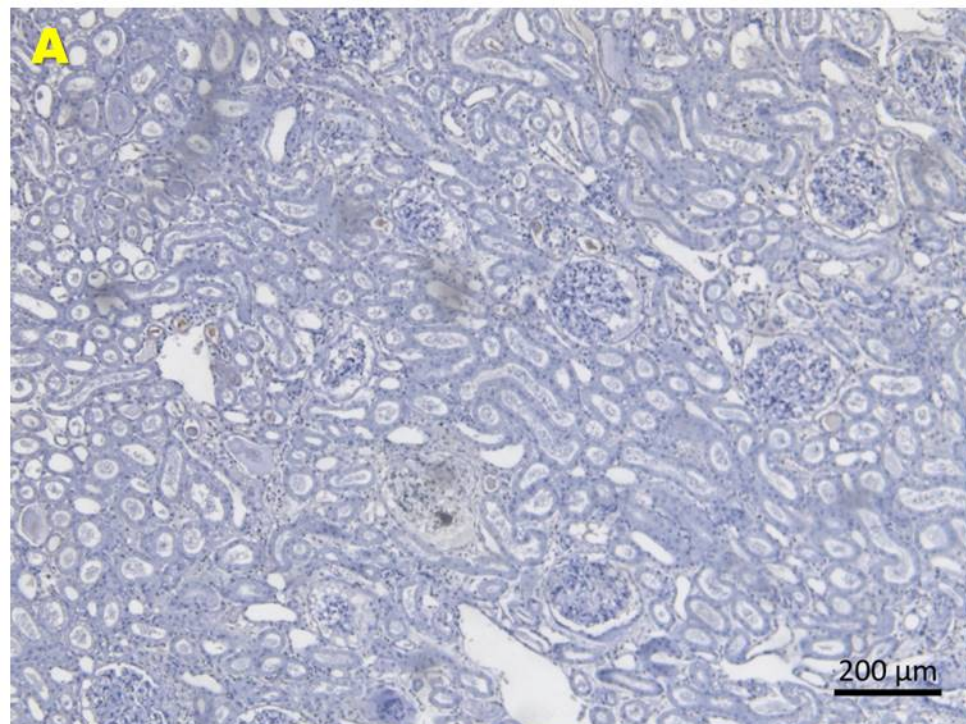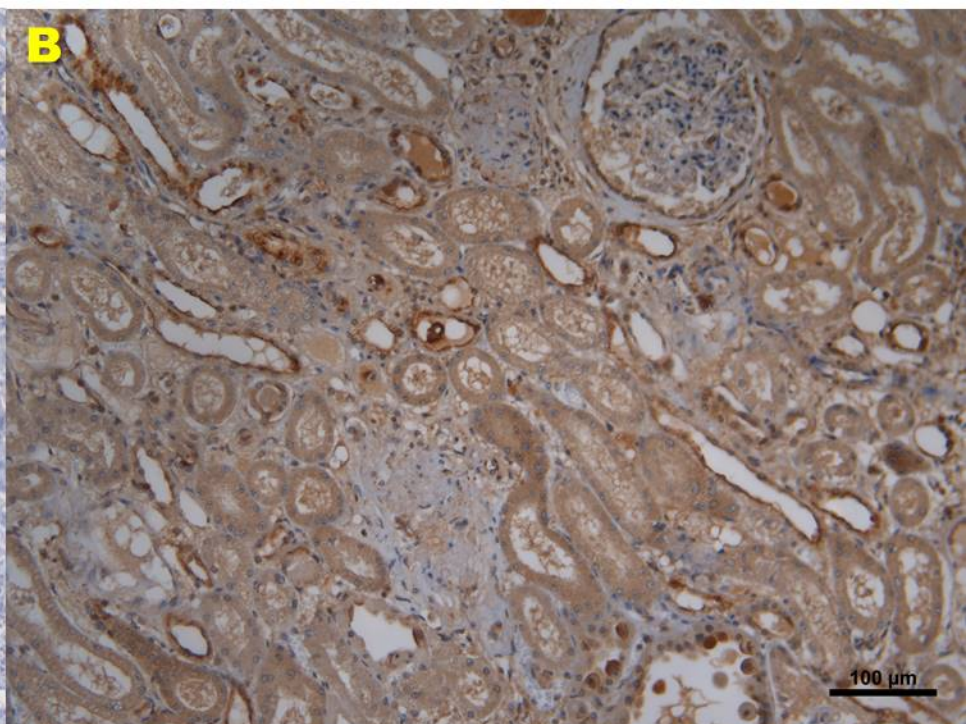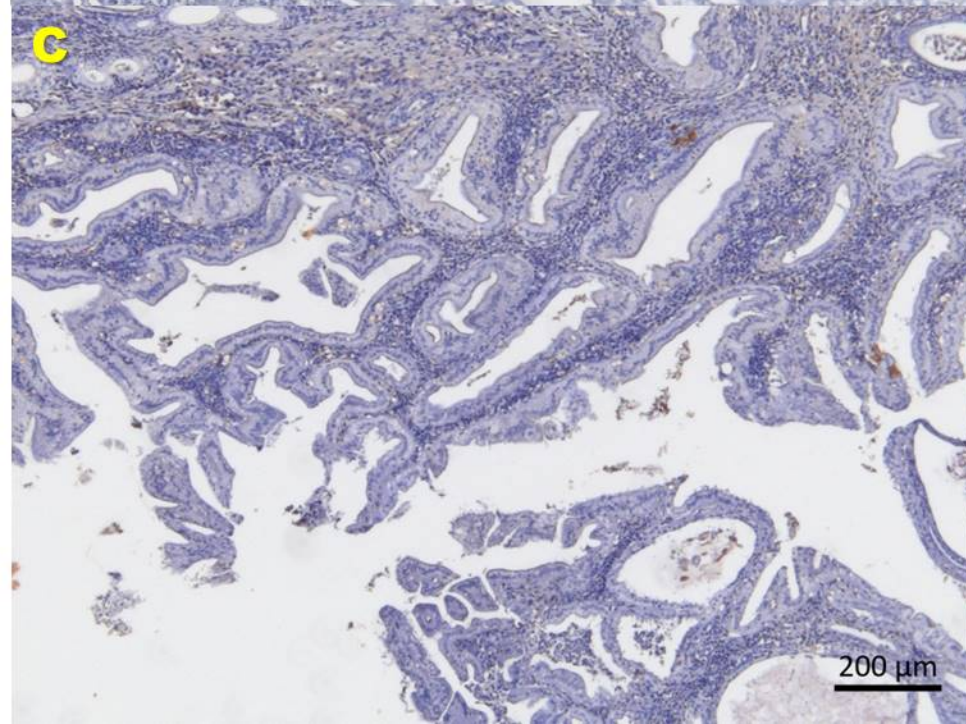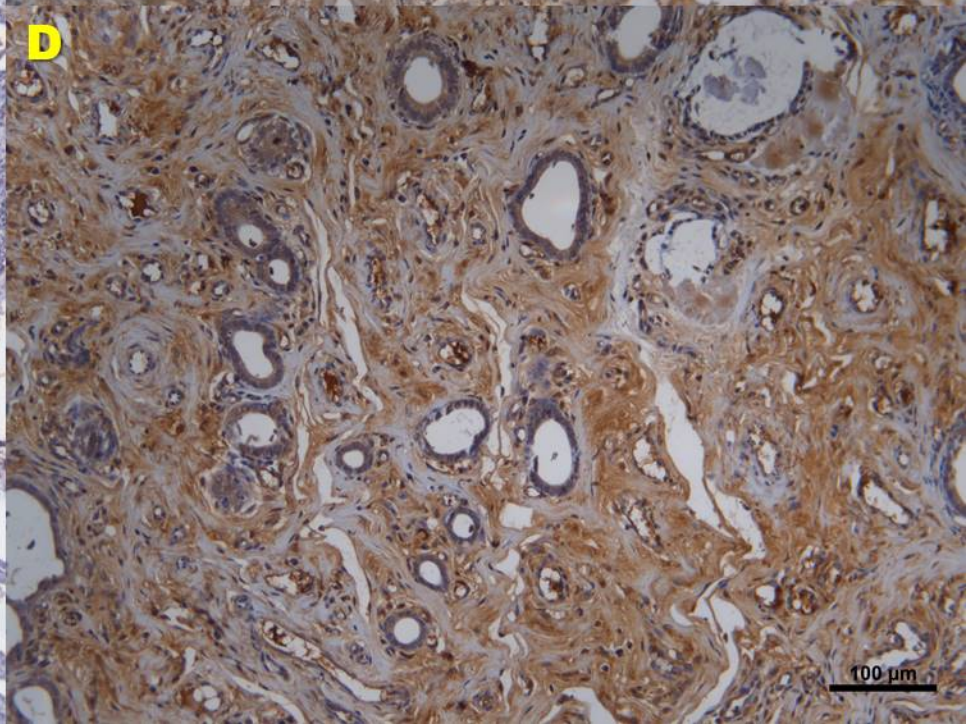

Supplement: Supplementary Figure 2. Immunostaining for eNOS (A) Negative control in the canine kidney, (B) Positive control in the canine kidney, (C) Negative control in the canine uterus, (D) Positive control in the canine uterus. 200x. [file supplementary_figure_2.pdf]
