# Supplementary material for: Prostaglandin and antigestagen in pyometra bitches: vascular and stereological effect
Source: Reprod Fertil. 2021 Apr 19;2(2):95–105. doi: 10.1530/RAF-20-0020 (PMC8812451; doi:10.1530/RAF-20-0020)
Supplement: Supplementary Table 1. VEGF-A and eNOS antibody characteristics and cross reactivity. [file supplementary_table_1.pdf]

Supplementary Table 1. VEGF-A and eNOS antibody characteristics and cross reactivity.

Antibody (Ref.)/CiteAb link	Experiment	Tissue/Cells	Reference
Anti-eNOS antibody (ab5589)<br>• CiteAb: https://www.citeab.com/antibodies/727893-ab5589-anti-enos-antibody?des=745f624045db3a11<br>• Labome: https://www.labome.com/product/Abcam/ab5589.html<br>• Datasheet: https://www.abcam.com/enos-antibody-ab5589.html	WB	Cardiac tissue (Left ventricle from golden retriever muscular dystrophy model)	Hammers et al., 2016
Anti-VEGFA antibody [VG-1] (ab1316)<br>• CiteAb: https://www.citeab.com/antibodies/759054-ab1316-anti-vegfa-antibody-vg-1?des=5bb43dae3b1d2922<br>• Labome: https://www.labome.com/product/Abcam/ab1316.html<br>• Datasheet: https://www.abcam.com/vegfa-antibody-vg-1-ab1316.html	Flow cytometry	Canine inflammatory mammary carcinoma cell line (IPC-366)	Borghesi et al., 2020
		Canine cochlear epithelial cells	Santos et al., 2017
	Immunocytochemistry and flow cytometry	Canine yolk sac cells	Fratini et al., 2016

References

Hammers, D.W.; Sleeper, M.M.; Forbes, S.C.; Shima, A.; Walter, G.A.; Sweeney, H.L. (2016). Tadalafil Treatment Delays the Onset of Cardiomyopathy in Dystrophin-Deficient Hearts. Journal American Heart Association. **5,** e003911. doi: 10.1161/JAHA.116.003911. PMID: 27506543; PMCID: PMC5015305.

Borghesi, J.; Caceres, S.; Mario, L.C.; Alonso-Diez, A.; Silveira Rabelo, A.C.; Illera, M.J.; Silvan, G.; Miglino, M.A.; Favaron, P.O.; Carreira, A.C.O.; Illera, J.C. (2020). Effects of doxorubicin associated with amniotic membrane stem cells in the treatment of canine inflammatory breast carcinoma (IPC-366) cells. BMC Veterinary Research. **16**, 353. doi: 10.1186/s12917-020-02576-0. PMID: 32972410; PMCID: PMC7513323.

Santos, A.C.; Borghesi, J.; Mario, L.C.; Anunciação, A.R.; Mess, A.M.; Carreira, A.C.; Favaron, P.O.; Miglino, M.A. (2017). Cochlear epithelial of dog fetuses: a new source of multipotent stem cells. Cytotechnology. **69**, 179-189. doi: 10.1007/s10616-016-0049-0. PMID: 28074389; PMCID: PMC5264633.
