# Supplementary material for: Prostaglandin and antigestagen in pyometra bitches: vascular and stereological effect
Source: Reprod Fertil. 2021 Apr 19;2(2):95–105. doi: 10.1530/RAF-20-0020 (PMC8812451; doi:10.1530/RAF-20-0020)
Supplement: Supplementary Table 2. Mean and standard error (X±SE) of the uterine artery [file supplementary_table_2.pdf]

Supplementary Table 2. Mean and standard error (X±SE) of the uterine artery hemodynamic analysis of Ovariohysterectomy (n=7), Aglepristone (n=5) and Associative Therapy (n=5) groups before the onset of treatment.

	Ovariohysterectomy	Aglepristone	Associative
Peak systolic velocity (PSV – cm/s)	58.08±5.4	74.44±7.5	79.28±7.5
End diastolic velocity (EDV – cm/s)	24.45±2.9	25.49±3.1	29.18±2.9
Time average maximum velocity (TAMAX – cm/s)	33.22±3.8	37.13±4.3	40.87±4.2
Peak systolic : diastolic velocity (S/D)	2.83±0.2	3.21±0.3	2.75±0.1
Resistance Index (RI)	0.61±0.02	0.65±0.03	0.62±0.02
Pulsatility Index (PI)	1.26±0.1	1.42±0.1	1.23±0.08
